# Taxonomical and functional analyses of epifaunal polychaetes associated with *Mussismilia* spp.: the effects of coral growth morphology

**DOI:** 10.7717/peerj.15144

**Published:** 2023-04-13

**Authors:** Marcos Nogueira, Wagner Magalhães, Eduardo Mariano-Neto, Elizabeth Neves, Rodrigo Johnsson

**Affiliations:** Universidade Federal da Bahia, Salvador, Bahia, Brazil

**Keywords:** Interaction, Association, Refuge, Habitat complexity, Habitat structure, Functional diversity

## Abstract

**Background:**

The increasing habitat heterogeneity and complexity shows positive effects over different communities, leading to environmental diversity, access to resources and reducing the effectiveness of predation. In the present study we evaluate the structural and functional patterns of polychaete assemblages of three *Mussismilia* species with different coral morphology. *Mussismilia hispida* has a massive growth pattern; *M. braziliensis* also is a massive coral but forms a crevice in the corallum base; and *M. harttii* has a meandroid pattern.

**Methods:**

Ten individuals of the three *Mussismilia* species were sampled in two reefs in the Todos-os-Santos Bay, and we analyzed the differences in richness and abundance of polychaete species and the functional diversity metrics: Rao’s quadratic entropy, functional dispersion, functional evenness, number of functional groups and functional richness, among *Mussismilia* species.

**Results:**

Two-way ANOVA with permutations showed significant differences for polychaete abundances and richness among *Mussismilia* species (higher values for *M. harttii*), but no differences were recorded when compared between the two coral reef areas studied. There was no statistical difference among coral species or between reefs in relation to the functional diversity components influenced by abundance, such as Rao quadratic entropy, functional dispersion, and functional evenness. Some individual polychaete functional traits presented differences among *Mussismilia* species, and that also helped us to build a picture about the effect of different growth structures over functional aspects of polychaete assemblages. Thus, the taxonomical approach, the analysis of individual functional traits and the functional diversity metrics are fundamental tools to characterize the assemblage of organisms associated with corals.

## Introduction

MacArthur & MacArthur (1961) were among the first to recognize the influence of habitat structure on animal’s diversity in different habitats. Since then, the number of studies concerning the effects of habitat structure in different environments have increased, including evaluations over other community attributes in addition to diversity (*e.g*., species abundance, species distribution, and richness) (Beck, 2000; Vytopil & Willis, 2001; Langellotto & Denno, 2004; Tews et al., 2004; Grabowski, Hughes & Kimbro, 2008; Carvalho & Barros, 2017).

The relationship between habitat structure and different communities can be summarized by the increase in habitat complexity and heterogeneity on the increment in the available niche spaces and increasing environmental diversity, facilitating the access to resources, and providing shelter from predators (Bazzaz, 1975; Menge & Sutherland, 1976; Vytopil & Willis, 2001; Piko & Szedlmayer, 2007).

The presence and abundance of organisms from coral reef systems may be dependent on coral species for various reasons, including food, shelter, and/or recruitment (Stella, Jones & Pratchett, 2010). Thus, the epifaunal abundance, species richness and composition may be influenced by the differences in the morphology of the coral host species (Vytopil & Willis, 2001; Stella, Jones & Pratchett, 2010; Nogueira, Neves & Johnsson, 2015; Nogueira et al., 2020). Many organisms may depend on corals for habitat and shelter, among these macrofaunal polychaetes are known for being highly diverse and abundant in different environments (Hutchings, 1998a; Hutchings, 1998b). Polychaetes contribute to the diversity and abundance patterns characterizing the benthic communities (Olsgard & Somerfield, 2000).

Polychaetes have long been used as good indicators of marine ecosystem health due to their high taxonomic diversity, different feeding habits and reproductive strategies (*e.g.*, Hutchings, 1998a; Hutchings, 1998b; Pearson & Rosenberg, 1978; Wilson, 1991). Since the conceptual model of polychaete feeding guilds by Fauchald & Jumars (1979) based on feeding type, mobility and buccal morphology, several authors have utilized and expanded this approach for environmental studies (*e.g*., Pagliosa, 2005; Cheung et al., 2008; Otegui, Brauko & Pagliosa, 2016) and a more recent revision was provided by Jumars, Dorgan & Lindsay (2015).

The integration of structural and functional analyses is extremely relevant to understand and identify important ecosystem functions, habitat resilience and redundancy (Van der Linden et al., 2012; Magalhães & Barros, 2011). These functional analyses are regarded as ecologically relevant for monitoring, management and conservation given that biological traits linked to ecological functions can be maintained even when species composition is altered (Bremner, Rogers & Frid, 2003). In marine environments, the role of marine invertebrate diversity in the ecosystem function is determined by their biological traits (Bremner, Rogers & Frid, 2006). The most studied and used polychaete traits are related to feeding characteristics, since they can add information to survey data beyond species names and abundances (Woodin, 1987). However, although feeding mechanisms are recognized as essential in determining differences between communities, biological trait analysis is considered to be more useful than the relative taxon composition and trophic group approaches (Bremner, Rogers & Frid, 2003) because it includes other traits such as attachment to the substrate, body form and mobility.

Functional Diversity (FD) is defined by Petchey & Gaston (2006) as “a component of biodiversity that generally concerns the range of things that organisms do in communities and ecosystems”. There are several indices created for measuring FD (*e.g.,* Rao’s quadratic entropy, based on the sum of pairwise distances between species weighted by relative abundance; Functional Richness, based on the convex hull volume; Functional Divergence, based on the species deviance from the mean distance to the center of gravity weighted by relative abundance (Mouchet et al., 2010) and they have been important in understanding ecosystem processes, resilience to environmental disturbance, and ecosystem services (*e.g.*, Petchey & Gaston, 2006; Villéger, Mason & Mouillot, 2008; Laliberté & Legendre, 2010).

**Figure 1:**
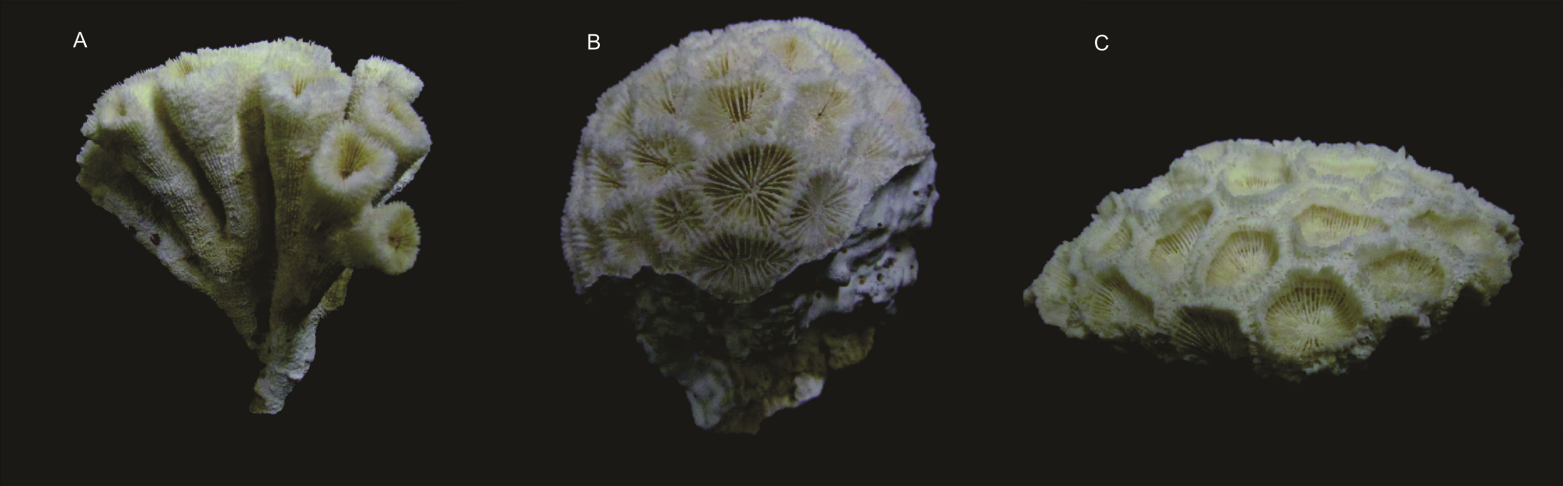
Morphological pattern of *Mussismilia* species. (A) *M. harttii*; (B) *M. braziliensis*; (C) *M. hispida*.

This study aimed to evaluate how the different morphological growth of three coral species of *Mussismilia* Ortmann, 1890, that represents difference in habitat structure for associated invertebrates, affects the structural and functional patterns of polychaete assemblages. The three *Mussismilia* species show different morphological patterns, characterized as a habitat structure gradient: *Mussismilia harttii* (Verrill, 1867) is the species that shows a more complex and heterogeneous structure, its polyps grow apart of each other generating spaces among them (meandroid pattern); *M. braziliensis* (Verrill, 1868) shows a massive growth pattern (the polyps grow together lacking space among them) with crevices at the corallum basis; and *M. hispida* (Verrill, 1901) that also shows a massive growth pattern, but the corallum basis is close to the substratum leaving no crevices (Fig. 1) (for more details see Nogueira, Neves & Johnsson, 2015). All three species are endemic to Brazil, representing common forms in almost all modern Brazilian reefs, and are among the six most important reef-building corals in Brazil (Laborel, 1970; Leão, Kikuchi & Testa, 2003).

## Materials & Methods

**Figure 2:**
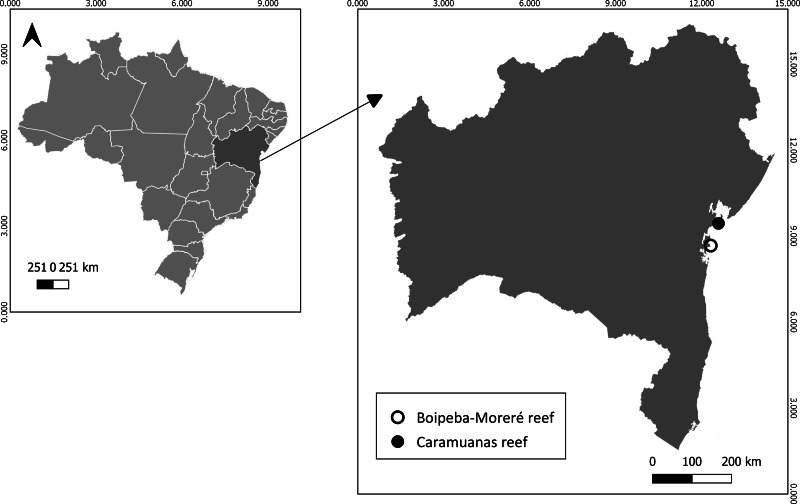
Sampling sites at Caramuanas and Boipeba Reefs in Bahia shore, northeastern Brazil.

The studied reefs were chosen for sampling due to the co-occurrence of all three species of *Mussismilia* in the Bahia state, Brazil: Caramuanas (13°70′S, 38°43′W) and Boipeba (13°28′S, 39°02′W) (Fig. 2). Both reefs are located within environmental protected areas. Caramuanas reef is located 4 Km from the coastal shore, the top of the reef is exposed during low tide, but blast fishing is common at the area with recorded reduction of some species as the hydrocoral *Millepora alcicornis* (Cruz, Kikuchi & Leão, 2009). The Boipeba reef belongs to the Tinharé-Boipeba Archipelago in the south shore of Bahia, also exposed during the low tide, but the tourism activity during the summer months is high due to the natural tide pools formed and to the close distance from the beach (Nogueira, Neves & Johnsson, 2015). Despite the status of protected area, there is no adequate environmental inspection.

Polychaete assemblages were examined in colonies of the three species of the genus *Mussismilia*. Coral samples were collected in February 2011 by scuba diving in depths varying from one to approximately four meters. In each reef, ten samples of each *Mussismilia* species were collected on the reef flat (corals with diameter between 15 and 25 cm), with a minimum distance of three meters between them, within an area of approximately 100 m^2^. The same coral species was never collected consecutively. All colonies were enclosed separately in plastic bags to avoid the escape of associated organisms and then removed from the substratum with a hammer and chisel.

In the laboratory, the corals were washed, the water was sieved through a 150 µm mesh and the organisms were stored in alcohol 70%. The polychaetes were sorted, identified, and counted under a stereomicroscope. Corals were bleached in a solution of 2.0% sodium hypochlorite before being deposited in the collection of the Natural History Museum of Bahia in the Federal University of Bahia (MHNBA, UFBA). Collecting permission was provided by the Chico Mendes Institute for Biodiversity Conservation (ICMbio) (Sisbio No 15161-1).

Functional diversity is the value and range of species traits influencing ecosystem functioning (Diaz & Cabido, 2001). However, traditional measures of FD are based only on the sum or the mean lengths of linear pair-wise distances between species, and do not include an important component of communities: the species abundance (Petchey & Gaston, 2006). In this way, Rao’s quadratic entropy seems to be a robust alternative, once it includes the abundance of species (Botta-Dukát, 2005). Therefore, this measure of FD is fundamental to comprehend the structure and functioning of communities.

The structural components of the polychaete assemblages used to access the taxonomical approach were the richness (number of species) and abundance (total number of individuals). The species were categorized into biological traits related to body size, feeding and reproductive aspects of their life history. Seven groups of traits showing 26 trait categories were chosen to represent polychaete functional diversity (Table 1). Polychaete feeding mode (*i.e.,* omnivore, carnivore, suspension feeder, surface deposit feeder, subsurface deposit feeder, and interface feeder), mobility while feeding (*i.e.,* motile, discretely motile, and sessile) and the morphological apparatus used for food collection (*i.e.,* tentacle/palps, muscular eversible pharynx, and non-muscular eversible pharynx) were selected based on Fauchald & Jumars (1979) and Jumars, Dorgan & Lindsay (2015). Additional traits are related to body size (total body length and total number of chaetigers), fate of ova (*i.e.,* free spawning, brooding on the outside of body, brooding inside the body, brooding inside tube, brooding of encapsulated embryos inside the tube, encapsulation of embryos in a gelatinous mass), type of larval development (*i.e.,* planktotrophic, lecithotrophic, direct benthic development) (Wilson, 1991), and type of asexual reproduction *i.e.,* stolonization, fragmentation, absent (Schroeder & Hermans, 1975).

**Table 1:** Biological trait variables and categories used to describe functional diversity in the polychates assemblage associated with *Mussismilia* species.

**Category**	**Trait**	**Function and processes (adapted from Beauchard et al., 2017)**
Body size	Total length (mm)	Fecundity increase, oxygen consumption, capacity to hide of predators
	Total number of chaetigers	
Feeding mode	Omnivore (O)	Food acquisition, growth requirements, nutrient cycling, particle transfer
	Carnivore (C)	
	Suspension feeder (S)	
	Surface deposit feeder (D)	
	Subsurface deposit feeder (B)	
	Interface feeder (I)	
Motility	Motile (M)	Foraging mode, ability to escape predation, dispersal, increase in habitat architecture (tubes)
	Discretely motile (D)	
	Sessile (S)	
Food delivered by	Tentacle/palps (T)	Removal of food items in bulk or individually
	Muscular eversible pharynx (P)	
	Non-muscular eversible pharynx (N)	
Assexual reproduction	Stolonization (ST)	Rapid habitat colonization, ensure demographic resilience in adversity or temporary dispersal
	Fragmentation (Frag)	
	Absent (ABS)	
Fate of ova	Free spawning (FS)	Juvenile survival and recruitment success
	Brooding on the outside of body (BR-EXT)	
	Brooding inside the body (BR-INT)	
	Brooding inside tube (BT-TUBE)	
	Brooding of encapsulated embryos inside the tube (BR-CAP)	
	Encapsulation of embryos in a gelatinous mass (GEL)	
Types of larval development	Planktotrophic (PLK)	Juvenile survival and dispersal potential
	Lecithotrophic (LEC)	
	Direct benthic development (DIR)	

Trait abundance from each coral reef and coral species was calculated as the mean value of each trait weighted by relative species’ abundances in each trait category (Garnier et al., 2004; Rumm et al., 2018). To evaluate the effects of coral species and reefs over FD components, we calculated Rao’s quadratic entropy, functional dispersion, functional evenness (the evenness of abundance distribution among species), number of functional groups (number of groups formed by traits association), and functional richness (number of different species functional traits) (Diaz & Cabido, 2001; Botta-Dukát, 2005; Petchey & Gaston, 2006; Villéger, Mason & Mouillot, 2008; Rumm et al., 2018). Gower distance was used to calculate traits by samples dissimilarity matrix, since we have quantitative and qualitative traits. Each FD component was then tested using two-way ANOVA tests, to deal with error distribution problems, the tests’ significance was calculated based on permutations.

All tests were performed in R environment (R Core Team, 2017). FD components were calculated with function dbFD of “FD” package (Laliberté & Legendre, 2010). Two-way ANOVA with permutation and Tukey’s post hoc tests were performed using the lmp function of “lmPerm” package (Wheeler & Torchiano, 2016).

## Results

**Figure 3:**
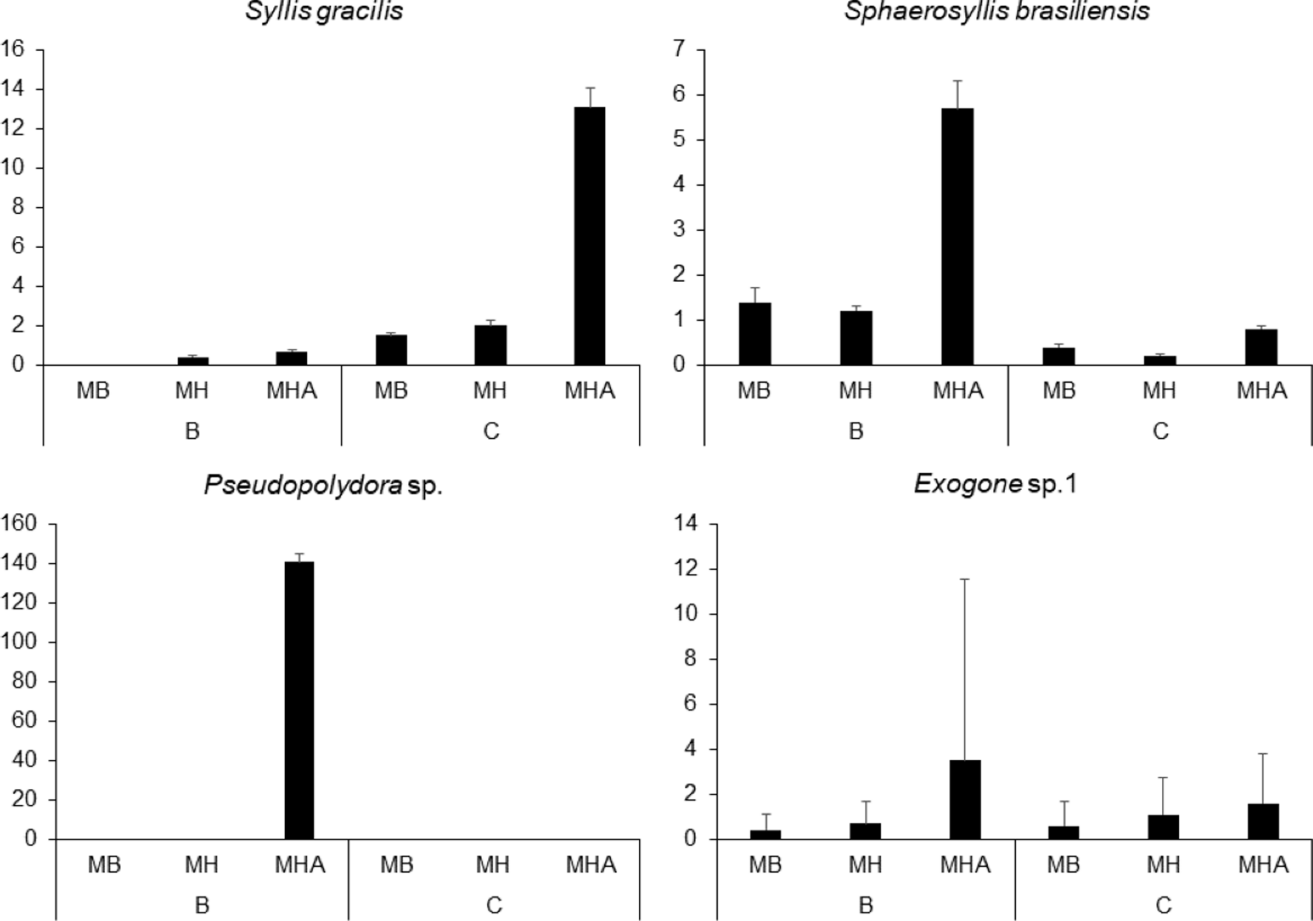
Abundance of the four most abundant polychaete species associated with *Mussismilia* species at Caramuanas and Boipeba reefs. MB, *M. braziliensis*; MH, *M. hispida*; MHA, *M. harttii*; B, Boipeba, and C, Caramuanas.

There were a total of 941 individuals in the samples from Caramuanas and Boipeba reefs. The most abundant species were the syllids *Syllis gracilis* Grube, 1840*, Sphaerosyllis brasiliensis* Nogueira, San Martín and Amaral, 2001*, Exogone* sp. and the spionid, *Pseudopolydora* sp.*,* comprising 43.7% of the total polychaeta abundance. All four most abundant species showed high densities in *M. harttii* colonies (Caramuanas or Boipeba), whereas the spionid *Pseudopolydora* sp. was only found in *M. harttii* colonies at Boipeba reef (Fig. 3).

**Figure 4:**
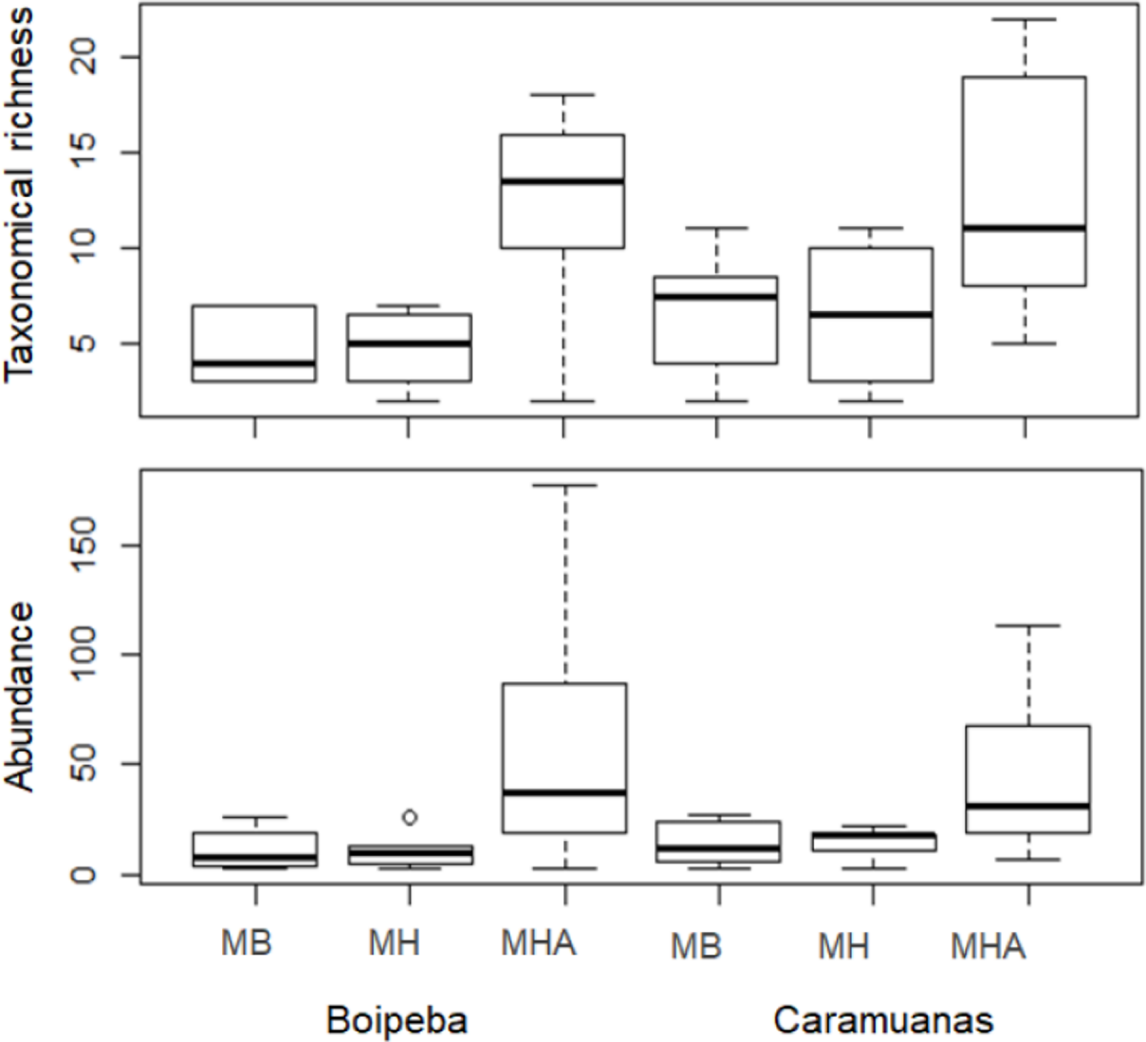
Comparisons of the taxonomical richness and abundance of polychaete species among *Mussismilia* species and between reefs. MHA, *M. harttii*; MB, *M. braziliensis*; MH, *M. hispida*.

Two-way ANOVA with permutation showed statistical differences for polychaete abundances among *Mussismilia* species, but no significant difference was recorded between the two coral reefs. The post hoc Tukey test found significant differences between polychaete assemblage within *M. harttii* and *M. braziliensis*, and between *M. harttii* and *M. hispida*, while no significant difference was recorded between *M. braziliensis* and *M. hispida*. For taxonomical richness, the same pattern was also observed in the post hoc test. In both reefs, *M. harttii* showed higher polychaete abundance and richness when compared to *M. braziliensis* and *M. hispida* (Fig. 4) (Table 2).

**Figure 5:**
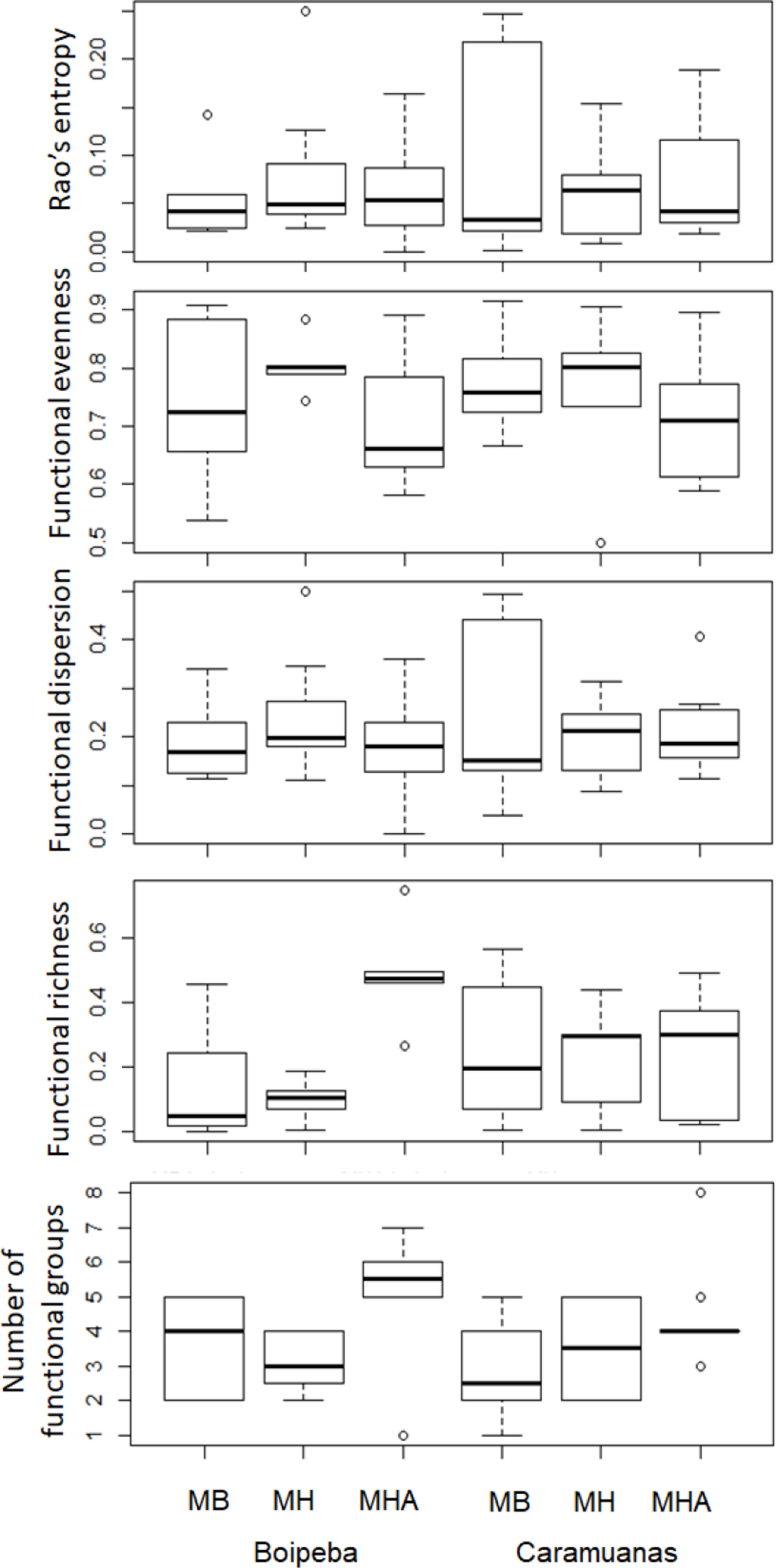
Comparisons of the Rao’s quadratic entropy (RaoQ), the Functional dispersion and the functional evenness among *Mussismilia* species and between reefs. MHA, *M. harttii*; MB, *M. braziliensis;* MH, *M. hispida*.

Regarding the selected components of the FD, we did not find significant differences among coral species or between reefs for the Rao quadratic entropy, functional dispersion, and functional evenness (Table 3) (Fig. 5). Statistical differences were found for the number of functional groups between *M. harttii* and *M. hispida*, and for the functional richness also between the same coral species. However, we found significant interaction effects between reef and *Mussismilia* species. Higher values of functional richness were found at Boipeba reef (Table 3) (Fig. 5).

**Table 2:** Results of ANOVA with Permutation and Tukey test of the taxonomical richness and abundance of polychates species among *Mussismilia* species (MHA –*M. harttii*, MB - *M. braziliensis* and MH - *M. hispida*) and between reefs.

**Abundance**	Df	F	*p*	Tukey test	*p*
Coral	2	81	0.0013^*^	MHA × MB	0.0291
Reef	1	0.0284	0.867	MHA × MH	0.0012
Coral × Reef	2	0.2637	0.7696	MB × MH	0.4787
**Taxonomical richness**					
Coral	2	113	0.00017^*^	MHA × MB	0.009
Reef	1	0.9967	0.32497	MHA × MH	0.00015
Coral × Reef	2	0.1051	0.90055	MB × MH	0.3507

**Notes.**

* Statistical significance.

**Table 3:** Results of the ANOVA with Permutation for the comparisons of the Rao’s quadratic entropy (RaoQ), the Functional dispersion and the functional evenness among *Mussismilia* species and between reefs.

**RaoQ**	Df	F	*p*	Tukey test	*p*
Coral	2	0.0566	0.9451		
Reef	1	0.207	0.652		
Coral × Reef	2	0.5378	0.5888		
**Functional dispersion**					
Coral	2	0.2281	0.7972		
Reef	1	0.099	0.7549		
Coral × Reef	2	0.5458	0.5842		
**Functional eveness**					
Coral	2	1.1063	0.3439		
Reef	1	0.0259	0.8733		
Coral × Reef	2	0.3556	0.7037		
**Number of functional groups**					
Coral	2	3.8424	^*^0.031	MHA × MB	0.1349
Reef	1	0.5105	0.4797	MHA × MH	^*^0.0319
Coral × Reef	2	0.5242	0.5966	MB × MH	0.7934
**Functional richness**					
Coral	2	3.9493	^*^0.03002	MHA × MB	0.3025
Reef	1	^*^0.02	0.8847	MHA × MH	^*^0.0238
Coral × Reef	2	4.2197	^*^0.02427	MB × MH	0.3969

**Notes.**

* Statistical significance.

**Figure 6:**
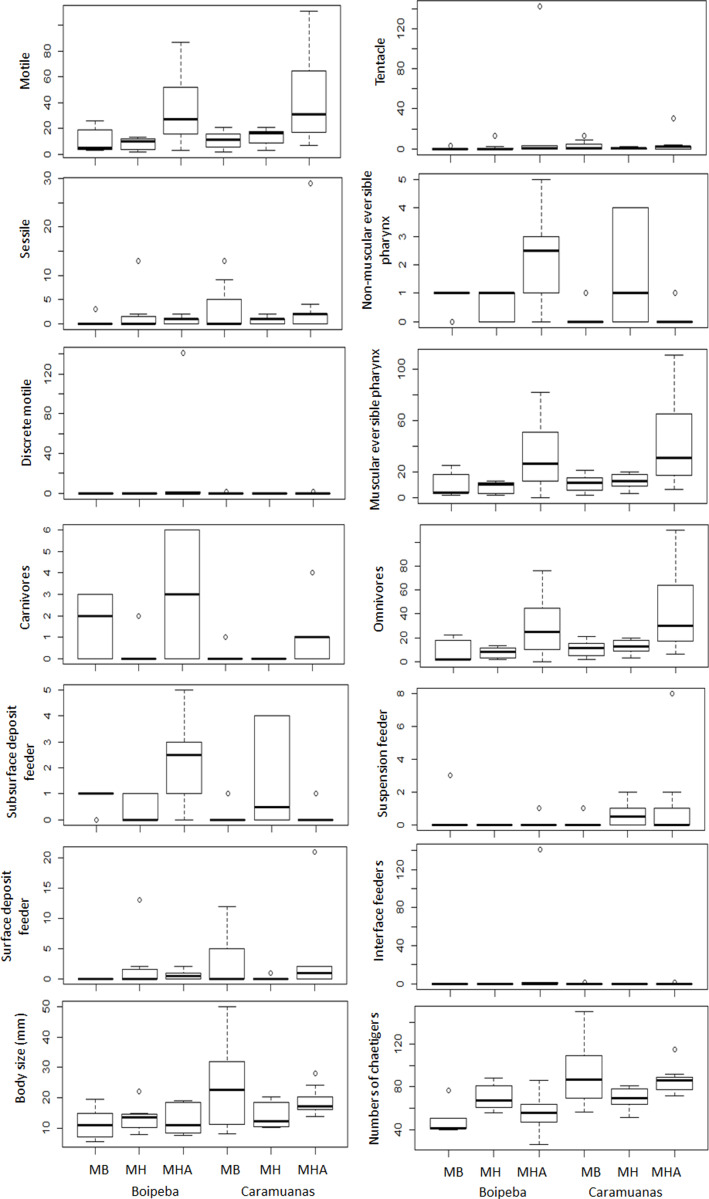
Comparisons of polychaete traits related to motility, mouth apparatus for food delivery, feeding mode and body size, among *Mussismilia* species and between reefs. MHA, *M. harttii*; MB, *M. braziliensis;* MH, M. hispida.

Analyzing each individual trait, we found significant differences among coral species for some feeding mode categories, but no differences between coral reefs. Considering omnivore, carnivores, and polychaetes with muscular eversible pharynx apparatus for food collection, *M. harttii* assemblages showed higher values in comparison with *M. braziliensis* and *M. hispida*, but no statistical differences were observed between the last two. However, we did not find differences in the abundance of all other feeding mode categories studied (*i.e.,* surface deposit feeder, suspension feeder, subsurface deposit feeder and interface feeders), and the tentacles and non-muscular eversible pharynxes morphological apparatus for food capture (Table 4) (Fig. 6).

**Table 4:** Results of the ANOVA with Permutation and Tukey test for the comparisons of polychaete traits related to motility, mouth apparatus for food delivery, feeding mode and body size, among *Mussismilia* species (MHA, *M. harttii*, MB, *M. braziliensis* and MH, *M. hispida*) and between reefs.

**Motile**	DF	*p*	Tukey test	*p*
Coral	2	^*^0.0008	MHA × MB	^*^0.0239
Reef	1	0.3057	MHA × MH	^*^0.0009
Coral × Reef	2	1	MB × MH	0.4668
**Sessile**				
Coral	2	0.9508		
Reef	1	0.6545		
Coral × Reef	2	0.697		
**Discrete motile**				
Coral	2	0.07078		
Reef	1	0.12394		
Coral × Reef	2	0.08564		
**Tentacle**				
Coral	2	0.06912		
Reef	1	0.35106		
Coral × Reef	2	0.35925		
**Non-muscular eversible pharynx**				
Coral	2	0.1704		
Reef	1	0.1001		
Coral × Reef	2	0.0008		
**Carnivores**				
Coral	2	^*^0.0054	MHA × MB	0.4505
Reef	1	^*^0.0038	MHA × MH	^*^0.0033
Coral × Reef	2	0.1674	MB × MH	0.0801
**Sub surface deposit feeders**				
Coral	2	0.2135		
Reef	1	0.0788		
Coral × Reef	2	0.051		
**Surface deposit feeders**				
Coral	2	0.9744		
Reef	1	0.8039		
Coral × Reef	2	0.7236		
**Omnivores**				
Coral		^*^0.0001	MHA × MB	^*^0.0284
Reef		0.5104	MHA × MH	^*^0.0013
Coral × Reef		0.8276	MB × MH	0.5047
**Suspension feeders**				
Coral		0.7931		
Reef		0.486		
Coral × Reef		0.4943		
**Interface feeders**				
Coral		0.09028		
Reef		0.36571		
Coral × Reef		0.10169		
**Body lenght**				
Coral		0.5169		
Reef		^*^0.0102		
Coral × Reef		0.1522		
**Number of chaetigers**				
Coral		0.9608		
Reef		^*^0.0001		
Coral × Reef		^*^0.0116		

**Notes.**

* Statistical significance.

Regarding trait categories related to body length, the general polychaete size (in mm) and number of chaetigers were similar across the *Mussismilia* species but showed statistical differences when the two reefs were compared. Polychaetes from the Caramuanas reef showed higher values of body length and number of chaetigers. On the other hand, traits related to mobility showed significant differences only for individuals with motile strategies. There were no statistical differences for sessile and discrete motile polychaetes. The abundance of motile individuals was higher in *M. harttii* assemblages when compared to the other *Mussismilia* species and no differences were verified between *M. braziliensis* and *M. hispida* (Table 4) (Fig. 6).

**Figure 7:**
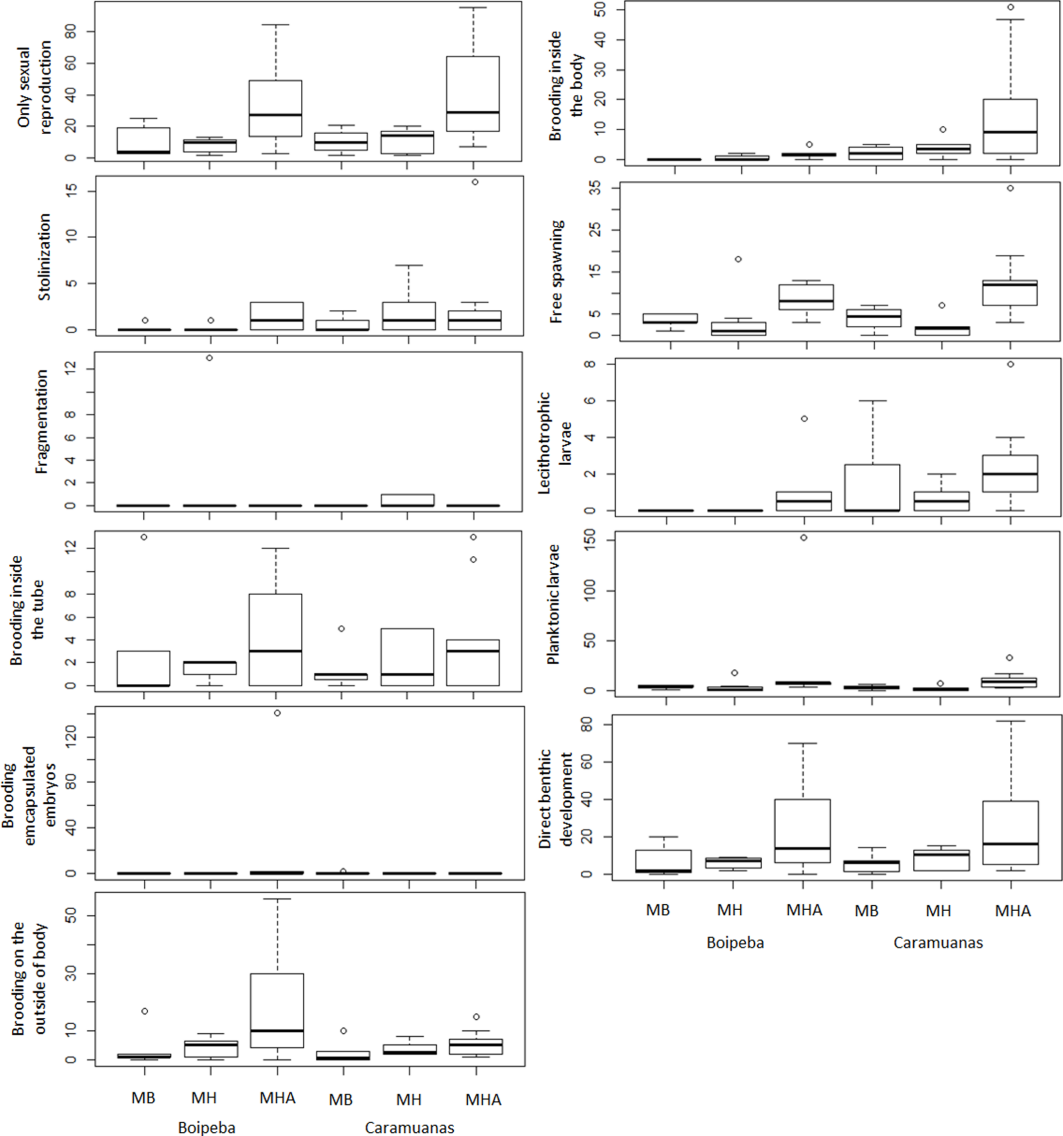
Comparisons of polychaete traits related to reproduction among coral species and between reefs. MHA, *Mussismilia harttii*; MB, *M. braziliensis;* MH, *M. hispida*.

The traits related to reproductive strategies showed no significant differences for the trait categories of asexual reproduction, both stolonization and fragmentation. Differences in abundance of the polychaetes that showed only sexual reproduction were observed among coral species assemblages. Higher values of asexually reproducing polychaetes were found in colonies of *M. harttii* when compared to *M. braziliensis* and *M. hispida*, and no difference was recorded between *M. braziliensis* and *M. hispida* (Table 5) (Fig. 7).

**Table 5:** Results of the ANOVA with Permutation and Tukey test for the comparisons of polychaete traits related to reproduction among coral species (MHA, *Mussismilia harttii*, MB, *M. braziliensis* and MH, *M. hispida*) and between reefs.

**Only sexual reproduction**	*p*	Tukey test	*p*
Coral	^*^0.0038	MHA × MB	^*^0.0024
Reef	0.4155	MHA × MH	^*^0.0013
Coral × Reef	0.9245	MB × MH	0.992
**Stolonization**			
Coral	0.3864		
Reef	0.217		
Coral × Reef	0.9468		
**Fragmentation**			
Coral	0.2251		
Reef	0.4296		
Coral × Reef	0.7625		
**Brooding inside the tube**			
Coral	0.1809		
Reef	0.8431		
Coral × Reef	0.7021		
**Brooding encapsulated embryos**			
Coral	0.4375		
Reef	0.6429		
Coral × Reef	0.623		
**Free spawning**			
Coral	^*^0.0004	MHA × MB	0.0636967
Reef	0.5051	MHA × MH	^*^0.001253
Coral × Reef	0.3554	MB × MH	0.307681
**Brooding inside the body**			
Coral	0.06201		
Reef	^*^0.02416		
Coral × Reef	0.20069		
**Brooding on the outside of the body**			
Coral	^*^0.0172	MHA × MB	0.116219
Reef	0.08566	MHA × MH	^*^0.03034
Coral × Reef	0.15181	MB × MH	0.822211
**Planktonic larvae**			
Coral	0.05236		
Reef	0.38509		
Coral × Reef	0.68786		
**Lecithotrophic larvae**			
Coral	^*^0.04091	MHA × MB	0.39668
Reef	^*^0.02858	MHA × MH	^*^0.03725
Coral × Reef	0.90196	MB × MH	0.44987
**Direct benthic development**			
Coral	^*^0.006	MHA × MB	0.062229
Reef	0.7843	MHA × MH	^*^0.008783
Coral × Reef	0.9608	MB × MH	0.712159

**Notes.**

* Statistical significance.

Reproductive traits related to egg releasing strategies and egg fate did not show statistical differences for polychaetes that brooded inside the tube, brooded encapsulated embryos and show planktonic larval release. Differences were observed between reefs for polychaetes that brood their young inside the body. Free spawning, brooding on the outside of body, the presence of lecithotrophic larvae and those polychaetes that undergo direct development represent reproductive traits that showed significant differences among coral species. These reproductive traits showed higher values in *M. harttii* assemblages when compared to *M. hispida*, with no additional differences in the other pairwise comparisons. We also identified differences between reefs for the use of lecithotrophic larvae, with higher values in the Caramuanas reef (Table 5) (Fig. 7).

## Discussion

The effects of habitat structure on the polychaete species composition are obvious among coral species with no influence of the reef in which they were sampled. The higher values of richness and abundance in *M. harttii* are in accordance with previous studies (Young, 1986; Nogueira, Neves & Johnsson, 2015; Nogueira et al., 2020). In fact, the meandroid morphology of *M. harttii*, with available space among corallites provides a more complex and heterogeneous habitat for the associated epifauna, acting as a refuge against predators (Nogueira, Neves & Johnsson, 2015; Nogueira, Neves & Johnsson, 2019). The massive growth pattern seems to be an unprofitable habitat for polychaetes, even in *M. braziliensis* that shows crevices at the base of the colony. The same pattern is observed in relation to the species abundance that was found in higher numbers in *M. harttii* corals. Polychaetes species are one of the most abundant macrozoobenthic group found associated with *Mussismilia* corals in Caramuanas and Boipeba, when compared to the total number of individuals found in previous studies: Decapoda (273 individuals) (Nogueira, Neves & Johnsson, 2015; Echinodermata (170) Nogueira et al., 2020); and Mollusca (495) (Nogueira, Neves & Johnsson, 2021).

Syllids are the most diverse and abundant polychaetes collected in all three species of *Mussismilia*. Given their active life style and generally small body size, they are able to move through crevices and burrows and are usually among the most abundant and diverse polychaetes found associated with sponges (Magnino et al., 1999; Neves & Omena, 2003), seaweed (Martins et al., 2013; Magalhães & Bailey-Brock, 2014), seagrass (Bone & San Martín, 2003), corals and hydrocorals (Martin & Britayev, 1998; Nogueira, San Martín & Amaral, 2001). Syllids have constantly been considered as generalist feeders but this may be due to the difficult in studying their feeding habits given their small size. Giangrande, Licciano & Pagliara (2000) observed diverse gut contents (*e.g*., fragments of algae, sponge spicules, detritus) suggesting a trophic niche separation among different species. *Syllis gracilis* and *Sphaerosyllis brasiliensis* were most abundantly collected at colonies of *M. hispida* from Caramuanas and Boipeba reefs, respectively. The material identified as *Syllis gracilis* may correspond to a different related species because there are increasing evidence that this species complex includes several cryptic and pseudo-cryptic lineages (*e.g.*, Álvarez Campos, Giribet & Riesgo, 2017; Langeneck et al., 2020). *Sphaerosyllis brasiliensis* was originally described from colonies of *M. hispida* from islands off the coast of São Paulo southeastern Brazil (Nogueira, San Martín & Amaral, 2001).

We found that the FD components that are correlated with abundance, such as functional dispersion, functional evenness and Rao’s quadratic entropy did not respond to the differences in the habitat structure provided by *Mussismilia* corals. Although, the components of the taxonomical approach (richness and abundance of species) did respond to the differences in habitat structure. The evaluation of the polychaete assemblages associated with *Mussismilia*, based on the analysis of its species and functional richness (number of different functional traits) indicates that the latter are relevant and informative (Diaz & Cabido, 2001), regarding ecosystem functions in the present study.

The protection of biogenic habitats may provide less variation in environmental severity when compared with more exposed habitats, such stability may benefit several species what outcome in higher numbers associated with biogenic habitats (Boyé et al., 2019). However, strong competitive interactions may arise from environmentally undisturbed sites (Defeo & McLachlan, 2005) that leads to high trait divergence among species coexisting at the same habitat (Perronne et al., 2017). In the *Mussismilia* genus, *M. harttii* is the species that creates higher complexity and heterogeneity, followed by *M. braziliensis*, providing shelter for higher number of species (Nogueira, Neves & Johnsson, 2015), In this way, the higher functional richness in *M. harttii* colonies of Boipeba reefs may reflect the role of shelter provided by *M. harttii* together with higher pristine conditions at Boipeba reef when compared to anthropogenic impacts as blast fishing recorded in Caramuanas reef (Cruz, Kikuchi & Leão, 2009).

We found differences for both, taxonomical and functional richness, metrics related to species richness of polychaetes among *Mussismilia* corals (higher values in *M. harttii* colonies from Boipeba reef). The conservation status of Boipeba reefs may contribute to pristine conditions for polychaetes, even under tourist visitation, *M. harttii* colonies were able to harbor more species, an event that promotes broader functional spaces (higher functional richness and dispersion) (Boyé et al., 2019).

According to Mason et al. (2005), when low functional richness is recorded, it indicates that some of the resources potentially available to the community may be unexplored, increasing the opportunity for invaders. Another outlook may be the absence or limited available resource, that restricts the occupation by other species. In this way, the meandroid growth morphology of *M. harttii* is a more complex habitat, when compared to the massive growth pattern of *M. braziliensis* and *M. hispida* (Nogueira, Neves & Johnsson, 2015). It seems that *M. harttii* provides easy access to exploitation of the resources, more available niches and/or protection against predation, when compared with the other *Mussismilia* species. This is also confirmed by the higher number of functional groups recorded in the present study associated with *Mussismilia harttii* colonies. In similar habitat conditions, the communities tend to show a high trait convergence among species (De Bello et al., 2010; Rumm et al., 2018).

Mason et al. (2005) also suggests a similar trend from the functional richness for functional evenness evaluation. The lower functional evenness observed suggests that some parts of niche space are under-utilized. However, the functional evenness did not differ among corals, suggesting that the trait abundance distribution is equivalent among *Mussismilia* species. Despite of this, the analysis of traits abundance individually indicates that all traits showed high abundance values associated with *M. harttii* colonies, except for carnivore, body length, number of chaetigers, brooding inside the body, and lecithotrophic larvae.

Studies based on the taxa composition approach discuss the function of species indirectly (post-analysis and only on selected taxa), commonly regarding feeding preferences and body size (Bremner, Rogers & Frid, 2003). Even if it incorporates some ecological information, this method is subjective, and it only allows a first insight into the functioning of the system. On the other hand, the trophic group approach considers ecological characteristics at the beginning of the analysis, but it is restricted to feeding traits limiting the ability to elucidate the community functional organization. The limited information provided by the previous approaches can be complemented by the biological trait analysis that directly incorporates a wide range of ecological characteristics. Biological traits are an important tool to measuring FD, as they are composed by phenotype characteristics of the individuals that may influence ecosystem level processes (Petchey & Gaston, 2006).

Studies of polychaetes assemblages concerning ecological evaluations are commonly supported by ecological indexes based on species composition. Several studies have included polychaete feeding guilds as conceptualized by Fauchald & Jumars (1979) to understand community structure and functioning (*e.g*., Cheung et al., 2008) but the most recent studies have added biological traits related to body size, habitat, and reproductive characteristics (*e.g*., Boström, Törnroos & Bonsdorff, 2010; Oug et al., 2012). In the present study, additional traits related to body size and reproductive characteristics (*i.e.,* fate of eggs, type of larval development, and type of asexual reproduction) were also considered.

As suggested by Bremner, Rogers & Frid (2003), biological trait analyses provide a complete assessment of benthic communities given that it is possible to identify ecosystem functions, in comparison to analyses based only on taxon composition or the trophic group approach. This comprehensive method has also helped understand the functional structure of estuaries (*e.g.*, Van der Linden et al., 2017), seagrass (Boström, Törnroos & Bonsdorff, 2010), sandy beaches (Wouters et al., 2018) and should be largely applied to other environments such as coral reefs.

Biological traits related to body size such as body length and total number of chaetigers were chosen for being considered important based on the expectation that the habitat selection for organisms associated with *Mussismilia* species could be strongly influenced by predation pressure (Nogueira, Neves & Johnsson, 2019). However, they did not show statistical significance among coral species. Considering that smaller individuals are more susceptible to predation than larger ones, it seems that in a more exposed habitat as the massive corals (*M. hispida* and *M. braziliensis*), smaller organisms would avoid it, or be easily predated, suggesting the importance of body size and number of chaetigers. Statistical differences were observed for body size traits when comparing both reefs. Other studies also considered the importance of body size but did not find it significant in differentiating the analyzed community such as the epibenthic megafauna subtidal community of coastal waters (10 to 50 m depth) studied by Bremner, Rogers & Frid (2003).

The difference found in the present study for body size traits could indicate that the Caramuanas reef may sustain a higher heterotrophic biomass through time (Chu et al., 2014), especially in relation to the abundances of eunicid and nereidid polychaetes. Other possible explanation may be related to reef characteristics. Differently from Boipeba Reef, that is located close to the beach and suffers intense touristic activity, Caramuanas Reef is 4 Km distant from the shore. The different conditions in which each reef is submitted may influence the occurrence of different species of predators for the polychaetes, bigger fishes may avoid Boipeba reef due to the touristic activities, allowing the growth of individuals associated with *Mussismilia* species in it.

Trait categories related to mobility have important roles in structuring benthic communities in stressful conditions, in which mobile individuals take advantage and increase in abundance (Bradshaw, Veale & Brand, 2002). The impact of predation over the invertebrates living associated with corals may act as a filter for mobility traits. Motile polychaetes showed higher abundance associated with *M. harttii* colonies and were likely attracted by the protection against predators as an avoidance mechanism, whereas sessile and discretely motile organisms did not showed difference among corals species, their establishment in the colony occurs during the settlement period, what restricts its dispersion to other colonies after that. Mobility in polychaetes is usually related to burrowing, crawling, and swimming movements (Jumars, Dorgan & Lindsay, 2015). Discretely mobile and sessile polychaetes are associated to burrow and tube construction and these are not facilitated on living substrates such as *Mussismilia* colonies.

Omnivore and carnivore polychaetes showed higher abundances in colonies of *M. harttii*. The more diverse morphology of *M. harttii* in comparison to the other two species may provide a broad spectrum of food items for polychaetes to explore. In comparison to soft-bottom substrates (*e.g*., Barroso, Paiva & Alves, 2002; Pagliosa, 2005; Magalhães & Barros, 2011), coral colonies do not favor the presence of deposit-feeding polychaetes. It may indicate that high quality food in low biomass is available in coral colonies as broadly omnivores such as eunicids, nereidids and syllids were the most abundant polychaete taxa. Suspension-feeders were also not abundantly found in living colonies of *Mussismilia* and these taxa, especially sabellids and serpulids, may have the feeding apparatus outcompeted by coral polyps.

Colonies of *M. harttii* also favored the presence of polychaetes that are free spawners, brood their young outside of body, and either produce lecithotrophic larvae or are direct developers. Most of these reproductive strategies are related to low dispersal potential and rapid colonization of newly occupied environments but local catastrophies may cause high extinction rates (McHugh & Fong, 2002).

The analysis based on taxonomical and FD approach done in the present study indicated that the FD metrics showing statistical difference are the ones tightly related with the taxonomical approach. Even higher number of species was found in *M. harttii* colonies, when the taxonomical richness effect is discounted using the FD metrics, the FD among the *Mussismilia* species is equivalent. Based on this, we suggest that the taxonomical approach and the analysis of individual traces, besides the use of functional diversity metrics, are a fundamental tool to better characterize the complexity of coral’s associate assemblages, and its responses to the environment.

##  Supplemental Information

10.7717/peerj.15144/supp-1 Supplemental Information 1 Raw data

## Additional Information and Declarations

### Field Study Permissions

The following information was supplied relating to field study approvals (i.e., approving body and any reference numbers): Collecting permission was approved by the Chico Mendes Institute for Biodiversity Conservation (ICMbio).

### Data Availability

The following information was supplied regarding data availability: The raw data is available in the Supplementary File.
